# Association of Glycemic Control With Different Diets Followed by Patients With Type 2 Diabetes: Findings From Qatar Biobank Data

**DOI:** 10.3389/fnut.2022.813880

**Published:** 2022-05-10

**Authors:** Reema Tayyem, Aya Hamdan, Karmen Alhmmadi, Yasmin Eissa, Maryam Al-Adwi, Zinab Al-Haswsa, Hiba Bawadi, Zumin Shi

**Affiliations:** Department of Human Nutrition, College of Health Sciences, Qatar University, Doha, Qatar

**Keywords:** diabetes mellitus (DM), HbA1c, glycemic control, diet, anthropometric measurements, dyslipidemia, hypertension

## Abstract

**Background:**

Diabetes mellitus type 2 (T2DM) is one of the most common diseases worldwide. Unhealthy dietary habits may lead to T2DM, which is also influenced by the extent of education and knowledge of appropriate diets for this disease.

**Aim:**

This study aims to highlight the possible association between following different types of diet (low-fat diet, low-calorie diet, low-salt diet, and more than one diet) and glycemic control among Qatari and long-term resident patients with T2DM.

**Methods:**

This study is secondary data analysis. Qatar Biobank (QBB) data on 2448 T2DM patients aged 18–60 years were obtained. The first group included participants with HbA1C <7%, while the second included those with HbA1c ≥ 7%.

**Results:**

The results of the association of glycemic control with different diets followed by patients with type 2 diabetes were adjusted in four models. In the 4th model (adjusted for gender, age, sociodemographic, anthropometric, dietary habits, comorbidities, and medications), results showed that poor patients with poor glycemic control have higher odds [OR 1.90; CI (1–3.63)] of following a low-salt diet. The same observation was found in the low-fat diet [OR 1.73; CI (1.06-3.07)]. However, patients following more than one diet showed lower odds of having poor glycemic control for about 32% [OR.69; CI (0.48–0.98)].

**Conclusion:**

Diet and lifestyle are vital factors that can affect HbA1C levels. The findings of this secondary analysis showed that better glycemic control of the patients was observed in patients following more than diet from the studied diets.

## Introduction

Diabetes mellitus (DM) is a group of metabolic diseases characterized by chronic hyperglycemia caused by one of two defects: a defect in insulin secretion or a defect in insulin action, or both (1). Several processes are involved in the development of diabetes, which causes autoimmune destructio*n* (at different levels) of β-cells in the pancreas, leading to insulin deficiency. Because insulin is an anabolic hormone, a lack of insulin in the target tissues results in abnormal carbohydrate, fat, and protein metabolism (2). The effect of insulin deficiency due to insufficient production of insulin / or the effect of decreased tissue response to insulin results at one or several points in the complex pathways of the hormone's action (2).

For several reasons, although type 2 diabetes mellitus (T2DM) is known to be widespread in adults, the incidence of this type has begun to increase among young adults. The most important reasons are the changes in their lifestyle when they were children, both in terms of life instability and the lower consumption of healthy foods. Obesity has been linked to insulin resistance (3). DM development is affected by uncontrollable factors such as age, gender, race, and genetics, as well as controllable factors that include diet, physical activity, and smoking. The dietary practices of people with DM are influenced by the extent of their education and knowledge of appropriate diets for T2DM (4). Therefore, nutritional intervention is an important and contributing factor in reducing the progression of the disease and preventing the emergence of complications related to type 2 diabetes. Studies revealed that the type and composition of the diet could play a role in the development and management of T2DM (5–7). The consumption of high amounts of fatty and sugary food would lead to obesity, which in turn may contribute to the risk of increased insulin resistance, especially if there is an accumulation of visceral fat (8). Consumption of vegetables and fruits, on the other hand, protects against T2DM development due to their high fiber and antioxidant content (9). Generally, following a low-carbohydrate or low-fat eating plan could contribute to the prevention of T2DM onset (10). Furthermore, following a very low-carbohydrate diet results in a long-term decrease in HbA1C levels (11), while following a low-salt diet can reduce high blood pressure, which is common in patients with T2DM (12). According to the National Academy of Medicine, medical nutrition therapy (MNT) is a treatment for a disease or condition that involves modifying nutrient or whole-food intake. In addition, the American Diabetes Associatio*n* (ADA) describes MNT as a fundamental component that must be integrated into the overall management plan for diabetes. Therefore, MNT is the primary intervention to achieve balanced and controlled blood sugar and avoid complications. Healthcare team members can accomplish these goals by providing evidence-based guidance to help patients with T2DM make healthy food choices and meet their own needs, not to mention that it is crucial to reassess them regularly during their treatment journey (10). The nutritional treatment plan varies from person to person depending on individual assessment of caloric requirements (based on age, weight, gender, and physical activity); personal preferences (such as culture, tradition, religion, health beliefs, economics), socioeconomic status, and metabolic goals to determine the most appropriate eating pattern for them (13).

Most of the studies demonstrated that there is an association between glycemic control and the consumption of several nutrients, such as simple carbohydrates, fats, and salt. However, there are few studies on eating patterns and their association with glycemic control (HbA1c <7.0) for Qatari adults (18–60) with diabetes. Therefore, our secondary analysis study aims to identify the possible association between glycemic control and different diets followed by Qatari adults with type 2 diabetes.

## Methods

### Study Population

Participants' data were obtained from Qatar Biobank (QBB) for secondary analysis. QBB is an initiative launched to make vital health research possible by gathering biological samples and information on the health and lifestyle of large numbers of people living in Qatar. The study population included 2,448 adults (females: 1,448; and males: 1,000) Qatari and long-term residents (individuals living in the country for ≥15 years) from 18 to 70 years of age with type 2 diabetes. Pregnant women and patients with terminating illnesses were excluded. Participants were classified into two groups: the first, which included T2DM participants with good glycemic control (HbA1c <7%), while the second group included participants with poor glycemic control (HbA1c ≥7%)] (14). Biochemical and anthropometric data were obtained from Qatar Biobank. It included weight (Kg), height (M), waist circumference, hip circumference, and HbA1c (%). Our study investigated the association between different types of diets: a low-fat diet, low-calorie diet, low-salt diet, and regular Qatari diet and glycemic control among people with T2DM living in Qatar. A qualitative food frequency questionnaire was used to provide data on how often participants consumed various foods and beverages and any modifications made to their diet over the preceding year. The detailed methods that were used in data sampling and collection have been published elsewhere (15). Qatar Biobank data collection and sample recruitment protocols were approved by the Hamad Medical Corporation Ethics Committee. The current analysis was approved under the IRB exempted category (QF-QBB-RES-ACC-00058).

### Independent Variable

Four types of diets were identified among the participants, including a low-fat dieta, low-calorie diet, low-salt diet, and regular diet. Additionally, many participants from the two groups followed more than one diet. The type of diet was determined based on the food choices and the participant's perception of his/her diet as compared to before diagnosis.

### Dependent Variable

- *Glycemic control* among patients with T2DM was considered the dependent variable for this study. HbA1c <7 was used as an outcome of the good glycemic control.
- *Anthropometric measurements*: body weight and height (sitting and standing) were measured using the Seca stadiometer and balance. Hip and waist circumferences were measured using tape. Body mass index (BMI) was calculated by dividing the weight (kg) by the height (m^2^) square.

### Covariates

Data such as age, education level, smoking status, and physical activity were obtained from participants using a self-administered health and lifestyle questionnaire. Education levels were divided into three categories: lower educatio*n* (up to secondary school), medium educatio*n* (technical or professional school), and higher educatio*n* (university and above). The physical activity levels were assessed in hours per week using the International Physical Activity Questionnaire. Face-to-face interviews with the patients were conducted with the assistance of professional nurses to gather information about their health status, related family medical history, and medication use. Health status (dyslipidemia and hypertension) was recorded from the patients' medical files.

### Statistical Analysis

Statistical Package for the Social Sciences version 23 (SPSS Inc., Chicago) was used to analyze the data. Data were presented as mean ± standard divisio*n* (SD) and frequency. Binary regressions were used to assess the association between glycemic control and diet, dietary habits, and sedentary behavior. This association was adjusted for four models. Model 1 is adjusted for gender, age, and comorbidities; model 2 for gender, age, sociodemographic, and comorbidities; model 3 for gender, age, sociodemographic, anthropometric, and comorbidities; and model 4 for gender, age, sociodemographic, anthropometric, dietary habits, and physical activity, comorbidities, and medications. The statistical significance level was set as *P*-value ≤0.05.

## Results

Table 1 shows the characteristics of the patients with HbA1c ≥ 7 % and patients with HbA1c <7 % by diet type: regular, low calorie, low-salt, and low-fat diet or more than one diet. In the group with HbA1c ≥ 7%, a significant difference was observed in the mean age of the patients following the first three diets as compared to those consuming a low-fat diet or more than one diet. In the group with HbA1c <7%, there was a difference between the mean ages within three different diets. While patients with HbA1c ≥ 7% on a low salt diet had the highest waist circumference mean, those on a low-fat diet had a significantly lower waist circumference compared to those on a low salt diet. There was a difference in the age group, and it was shown that most patients who follow the different types of diets mentioned in the study were 51–70 years old. The highest percentage of participants in different types of diets earned a university degree. Most of the patients (>60%) in the two groups reported high blood cholesterol, while 40–70% of these patients reported high blood pressure. Table 2 shows the different dietary habits and sedentary behavior of the study sample. The groups that follow a low-calorie diet, a low-fat diet or more than one diet in both groups mostly modified their diet in the last year, while most of the patients who follow a regular diet did not modify their diet in the last year. During the past year, most of the patients in the group with HbA1c ≥ 7 % following different types of diets ate from a common plate every day except those following a low-fat diet shared their plates 1–3 times/week. In the group with HbA1c <7%, those following low-calorie and more than one diet shared their plates 1–3 times/week. Additionally, differences in eating snacks between meals were observed. Most of the patients on the low-calorie, low salt or more than one diet consumed 3–5 times per week snacks between meals. In both groups, significant differences in the time spent on computers at weekends between different types of diets were observed. Patients, who follow a regular diet had the highest values, while the lowest values were observed in patients who follow a low-salt diet. In the group with HbA1C level ≥7%, 29.4 % of the participants following a low-fat diet spent <1 h on computers at weekends. Table 3 shows the association between the different types of diets and other variables with glycemic control. In the four adjusted models, the likelihood of poor glycemic control may increase when following a low-fat diet. Moreover, following a low-salt diet may increase the risk of having poor glycemic control of about 75% (OR 1.75; CI (1.18–3.14) in the third model (adjusted for gender, age, sociodemographic, anthropometric, and comorbidities) and 90% [OR 1.90; CI (1–3.63)] in the fourth model (adjusted for gender, age, sociodemographic, anthropometric, dietary habits, comorbidities, and medications). On the other hand, following more than one diet may improve glycemic control by 62–64% in all the adjusted models. Additionally, not modifying the diet increased the odds of having an HbA1C level significantly ≥7% for about 16% (OR: 1.16; 95 % CI; 1.07–1.43).

**Table 1:** Characteristics of the study sample by diet type.

**Variable**	**Patients with HbA1C ≥7%**					**Patients with HbA1C < 7%**				
	**Regular** **(*n =* 959)**	**Low-calorie diet ** **(*n =* 162)**	**Low-salt diet ** **(*n =* 89)**	**Low-fat diet(*n =* 169)**	**More than one diet ** **(*n =* 127)**	**Regular** **(*n =* 847)**	**Low-calorie diet (*n =* 182)**	**Low-salt diet ** **(*n =* 92)**	**Low-fat diet** **(*n =* 204)**	**More than one diet ** **(*n =* 133)**
	**Mean ±SD**					**Mean ±SD**				
Age (year)	53.4 ± 11.2	51.5 ± 11.4	54.4 ±12.1	52.9 ±13.7	52.7 ±12.8	50.1 ± 12.	47.8 ± 10.7	55.2 ± 12.8	51.7 ± 11.0	52.1 ±11.7
Weight (Kg)	85.0 ±16.3	86.0 ±20.1	89.0 ±17.3	81.8 ±13.3	84.8 ±17.1	82.8 ±6.2	84.1 ± 15.0	77.9 ± 13.1	82.6 ±12.2	80.9 ±13.7
Height (cm)	161.9 ±9.4	161.0 ±9.3	162.2 ± 10.5	163.0 ±9.3	164.3 ± 9.9	161.2 ± 9.3	161.6 ±9.7	158.0 ± 6.6	160.7 ±8.5	163.0 ±9.5
Body mass index (Kg/m^2^)	32.5 ±6.0	33.1 ±6.7	33.6 ± 4.1	30.9 ±5.0	31.3 ± 5.5	32.0 ±6.1	32.2 ±5.3	31.3 ± 5.5	32.1 ±4.7	30.5 ±5.0
Waist Circumference (cm)	99.8 ±12.2	99.6 ±15.1	103.8 ± 13.5	96.6 ±12.4	99.5 ±13.7	95.1 ±12.5	95.0 ± 12.3	91.7 ± 13.2	94.7 ±11.8	94.2 ±11.3
Hip Circumference (cm)	109.4 ±12.2	110.3± 13.1	112.1 ±9.3	107.4 ±9.9	107.9 ± 13.1	109.7 ± 12.1	110.3 ± 10.8	107.0 ± 9.1	110.2 ±10.3	107.5 ±9.9
HbA1C %	8.8 ±1.6	8.6 ±1.4	8.6 ±1.8	8.5 ±1.2	8.7 ±1.7	6.1 ±0.6	6.0 ±0.5	5.6 ± 1.1	6.1 ±0.6	6.1 ±0.6
	***n*** (%)^*^					***n*** (%)^*^				
**Age group (year):**										
•18–30	33 (3.4)	12 (7.4)	4 (4.5)	16 (9.5)	12 (9.4)	57 (6.7)	12 (6.6)	5 (5.4)	9 (4.4)	7 (5.3)
•31–50	322 (33.6)	50 (30.9)	17 (19.1)	39 (23.1)	28 (22.0)	351 (41.2)	86 (47.3)	25 (27.2)	82 (40.2)	50 (37.6)
•51–70	552 (57.6)	92 (56.8)	61 (68.5)	103 (60.9)	80 (63.0)	413 (48.5)	78 (42.9)	57 (62)	103 (50.5)	69 (51.9)
•70	52 (5.4)	8 (4.9)	7 (7.9)	11 (6.5)	7 (5.5)	31 (3.6)	6 (3.3)	5 (5.4)	10 (4.9)	7 (5.3)
**Gender**										
•Male	432(45.0)	83(51.2)	49(55.1)	89(52.7)	73(57.5)	306(35.9)	64(35.2)	46(50)	76(37.3)	56 (42.7)
•Female	527(55.0)	79(48.8)	40(44.9)	80(47.3)	54(42.5)	546(64.1)	118(64.8)	46(50)	128(62.7)	75 (57.3)
**Smoking**										
•No, have never smoked	650 (67.8)	97 (59.9)	52 (58.4)	98 (58)	69 (54.3)	600 (70.4)	125 (68.7)	54 (58.7)	127 (62.3)	78 (59.5)
•Yes, on most or all days	85 (8.9)	8 (4.9)	7 (7.9)	13 (7.7)	10 (7.9)	71 (8.3)	10 (5.5)	9 (9.8)	18 (8.8)	12 (9.2)
•Yes, only occasionally	34 (3.5)	13 (8.0)	5 (5.6)	11 (6.5)	9 (7.1)	23 (2.7)	16 (8.8)	5 (5.4)	9 (4.4)	10 (7.6)
•No, stopped smoking	107 (11.2)	25 (15.4)	17 (19.1)	26 (15.4)	23 (18.1)	69 (8.1)	14 (7.7)	9 (9.8)	14 (6.9)	10 (7.6)
**Educational level:**										
•Primary school and below	242(25.7)	16(9.9)	13(14.6)	24(14.3)	12(9.7)	151(18.1)	17(9.3)	13(14.1)	32(15.7)	15(11.6)
•Secondary and High school	200(21.2)	32(19.7)	21(23.6)	30(17.8)	25(20.4)	159(19.0)	29(15.9)	17(18.5)	35(17.2)	26(20.2)
•Technical or professional school	73 (7.8)	14 (8.6)	10 (11.2)	20 (11.8)	15 (12.2)	64 (7.7)	26 (14.3)	9 (9.8)	21 (10.3)	18 (14.0)
•University and Postgraduate degree	267(28.4)	62(38.3)	28(31.4)	60(35.5)	47(38.2)	288(34.5)	69(37.9)	36(39.1)	82(40.2)	52(40.3)
**Monthly Income (QR per month)**										
• <20,000	380(39.7)	58(35.8)	28(31.5)	62(36.7)	43(38.1)	324(38.0)	66(36.2)	36(39.1)	79(38.7)	49(39.8)
•20,001–80,000	329(34.4)	72(43.4)	42(47.2)	67(39.7)	52(46.0)	316(37.1)	81(44.5)	37(40.2)	80(39.2)	53(43.1)
•80,000<	62 (6.5)	11(6.8)	8 (9.0)	18 (10.7)	14 (12.4)	55 (6.5)	15 (8.2)	11 (12)	18 (8.8)	15 (12.2)
•No income	69 (7.2)	8 (4.9)	4 (4.5)	6 (3.6)	4 (3.5)	58 (6.8)	9 (4.9)	3 (3.3)	14 (6.9)	6 (4.9)
**Do you have high blood cholesterol?**	631(65.8)	108(66.7)	74(83.1)	127(75.1)	100(78.7)	498(58.5)	106(58.2)	63(68.5)	135(66.2)	87(66.4)
**Do you have high blood pressure?**	(44.9)430	71(43.8)	64(71.9)	86(50.9)	72(56.7)	(39.0)332	75(41.2)	58(63.0)	96(47.1)	72(55.0)
**Medications**										
•Tablets	744(77.6)	121(74.7)	70(78.7)	132(78.1)	101(79.5)	544(63.8)	106(58.2)	59(64.1)	127(62.3)	83(62.4)
•Insulin	374(39.0)	70(43.2)	45(50.6)	73(43.2)	58(45.7)	81(9.5)	20(11.0)	12(13.0)	24(11.8)	15(11.3)

* *The uncompleted 100 is due to the unspecified responses*.

**Table 2:** Dietary habits and sedentary behavior of the study sample.

**Variables**	**Patients with HbA1C ≥7%**					**Patients with HbA1C <7%**				
	**Regular** **(*n =* 959)**	**Low-calorie diet ** **(*n =* 162)**	**Low-salt diet** **(*n =* 89)**	**Low-fat diet** **(*n =* 169)**	**More than one diet** **(*n =* 127)**	**Regular** **(*n =* 847)**	**Low-calorie diet ** **(*n =* 182)**	**Low-salt diet** **(*n =* 92)**	**Low-fat diet** **(*n =* 204)**	**More than one diet ** **(*n =* 133)**
	***n*** (%)^*^					***n*** (%)^*^				
**Diet modification within last year**	223(23.20)	119(73.5)	62 (69.7)	125 (74)	90(70.90)	273(31.90)	143(78.6)	65 (70.7)	148 (72.5)	100(75.20)
**How often during a typical week in the last year did you eat from a common plate, shared with others?**										
•Every day	350 (36.5)	38 (23.5)	24 (27)	40 (23.7)	34 (26.8)	301 (35.6)	38 (20.9)	16 (17.4)	41 (20.1)	26 (19.5)
•>3 times/week	141 (14.7)	32 (19.8)	17 (19.1)	32 (18.9)	21 (16.5)	118 (14.0)	34 (18.7)	15 (16.3)	38 (18.6)	24 (18.0)
•1-3 times /week	140 (14.6)	31 (19.1)	14 (15.7)	39 (23.1)	26 (20.5)	155 (18.3)	57 (31.3)	21 (22.8)	46 (22.5)	31 (23.3)
•1/month	98 (10.2)	24 (14.8)	13 (14.6)	23 (13.6)	16 (12.6)	94 (11.1)	16 (8.8)	15 (16.3)	32 (15.7)	19 (14.3)
•Never or rarely	229 (23.9)	37 (22.8)	21 (23.6)	35 (20.7)	30 (23.6)	177 (20.9)	37 (20.3)	25 (27.2)	47 (23)	33 (24.8)
**How often, during a typical week in the last year, did you eat food from home-delivery, take-away, or fast-food restaurants?**										
• <1/week	220 (23.0)	37 (22.8)	14 (15.7)	38 (22.5)	25 (19.7)	181(21.4)	49 (26.9)	26 (28.3)	54 (26.5)	37 (27.8)
•1–2/week	176 (18.4)	30 (18.5)	9 (10.1)	27 (16.0)	20 (18.0)	178 (21.0)	40 (22.0)	15 (16.3)	40 (19.6)	28 (21.1)
•3–5 times/week	76 (7.9)	11 (6.8)	6 (6.7)	8 (4.7)	8 (3.6)	81 (9.6)	10 (5.5)	5 (5.4)	11 (5.4)	7 (5.3)
•Every day or almost everyday	16 (1.7)	3 (1.9)	1 (1.1)	1 (0.6)	1 (0.8)	32 (3.8)	2 (1.1)	1 (1.1)	2 (1.0)	1 (0.8)
•Never or rarely	470 (49.1)	81 (50.0)	59 (66.3)	95 (56.2)	73 (57.5)	375 (44.3)	81 (44.5)	45 (48.9)	97 (47.5)	60 (45.1)
**How often of eating snacks between meals in typical week in the last year (Meals: breakfast, lunch, and dinner)**										
•>7 times / week	118 (12.3)	26 (16.0)	10 (11.2)	22 (13.0)	18 (14.2)	121 (14.3)	27 (14.8)	10 (10.9)	23 (11.3)	13 (9.8)
•6–7 times per week	168 (17.5)	25 (15.4)	14 (15.7)	29 (17.2)	21 (16.5)	134 (15.8)	29 (15.9)	13 (14.1)	36 (17.6)	26 (19.5)
•One or twice per week	259 (27.0)	32 (19.8)	21 (23.6)	38 (22.5)	25 (19.7)	223 (26.3)	31 (17.0)	18 (19.6)	44 (21.6)	24 (18.0)
•3-5 times a week	182 (19.0)	39 (24.1)	22 (24.7)	44 (26.0)	33 (26.0)	152 (17.9)	61 (33.5)	29 (31.5)	58 (28.4)	43 (32.3)
•Prefer not to answer	232(24.2)	40 (24.7)	22 (24.7)	36 (21.3)	30(23.6)	217(25.6)	34 (18.7)	22 (23.9)	43 (21.1)	27(20.3)
**The level of activity involved:**										
•Sitting most of the time	417 (45.7)	58 (36.9)	27 (31.8)	57 (35.6)	42 (8.3)	341 (41.1)	76 (43.4)	33 (37.1)	83 (41.9)	55 (11.9)
•Standing most of the time	17 (1.9)	4 (2.5)	1 (1.2)	3 (1.9)	2 (8.0)	13 (1.6)	6 (3.4)	2 (2.2)	2 (1.0)	3 (15.8)
•Walking most of the time	36 (3.9)	14 (8.9)	4 (4.7)	12 (7.5)	9 (15.8)	33 (4.0)	6 (3.4)	4 (4.5)	13 (6.6)	5 (10.2)
•Sitting, standing, and walking in equal amounts	346 (37.9)	67 (42.7)	44 (51.8)	75 (46.9)	54 (11.9)	350 (42.2)	63 (36.0)	40 (44.9)	76 (38.4)	50 (10.9)
•Other work with moderate physical activity (includes moving or lifting objects of moderate weight)	59 (6.5)	12 (7.6)	7 (8.2)	9 (5.6)	9 (12.2)	63 (7.6)	19 (10.9)	5 (5.6)	14 (7.1)	9 (10.0)
•Physically heavy work (includes moving or lifting heavy objects or activities)	4 (0.4)	1 (0.6)	1 (1.2)	3 (1.9)	2 (28.6)	7 (0.8)	3 (1.7)	2 (2.2)	4 (2.0)	3 (25.0)

* *The uncompleted 100 is due to the unspecified responses*.

**Table 3:** Association between types of diets and other variables with glycemic control based on HbA1c.

**Variables**	**OR(95%CI)**	***P*-value**
**Binary logistic regression (model 1: adjusted for gender, age and**		
**comorbidities)**		
Gender	1.56(1.32–1.84)	<0.001
Age	0.98(0.97–0.98)	<0.001
Regular diet	1.00(0.67–1.49)	1
Low-calorie diet	1.34(0.84–2.14)	0.22
Low-salt diet	1.67(0.94–2.98)	0.08
Low-fat diet	1.86(1.11–3.11)	0.02
More than one diet	0.67(0.49–0.92)	0.01
Dyslipidemia	1.00(1.00–1.00)	0.41
Hypertension	1.00(1.00–1.00)	0.53
Constant	1.47	0.19
**Binary logistic regression (model 2: adjustedfor, age, gender**,		
**sociodemographic, and comorbidities)**		
Gender	1.69(1.384–2.065)	<0.001
Age	0.98(0.974–0.989)	<0.001
Regular diet	1.03(0.688–1.543)	0.89
Low-calorie diet	1.33(0.834–2.133)	0.23
Low-salt diet	1.68(0.940–2.993)	0.08
Low-fat diet	1.85(1.103–3.107)	0.02
More than one diet	0.67(0.48–0.92)	0.01
Dyslipidemia	1.00(1.00–1.00)	0.42
Hypertension	1.00(1.00–1.00)	0.53
Income	1.00(1.00–1.00)	0.67
Educational level	1.07(1.03–1.12)	<0.001
Smoking	1.01(0.94–1.10)	0.74
Constant	0.7	0.35
**Binary logistic regression (model 3: adjusted for age, gender**,		
**sociodemographic, anthropometric, and comorbidities)**		
Gender	1.83(1.49–2.25)	<0.001
Age	0.98(0.97–0.99)	<0.001
Regular diet	1.03(0.69–1.54)	0.89
Low-calorie diet	1.33(0.83–2.13)	0.24
Low-salt diet	1.75(1.18–3.14)	0.05
Low-fat diet	1.81(1.07–3.03)	0.03
More than one diet	0.66(0.48–0.91)	0.01
Dyslipidemia	1.00(1.00–1.00)	0.38
Hypertension	1.00(1.00–1.00)	0.52
Educational level	1.07(1.02–1.11)	<0.001
Income	1.00(1.00–1.00)	0.72
BMI	0.98(0.96–0.99)	0
Smoking	1.02(0.94–1.10)	0.64
Constant	1.29	0.57
**Binary logistic regression (model 4:adjusted for age, gender**,		
**sociodemographic, anthropometric, dietary habits and physical activity**		
**OR lifestyle habits, comorbidities, and medications)**		
Gender	1.96(1.56–2.45)	<0.001
Age	0.99(0.98–1.00)	0.1
Regular diet	1.07(0.68–1.69)	0.76
Low-calorie diet	1.30(0.77–2.20)	0.32
Low-salt diet	1.90(1.00–3.63)	0.05
Low-fat diet	1.73(1.06–3.07)	0.05
More than one diet	0.69(0.48–0.99)	0.04
Dyslipidemia	1.00(1.00–1.00)	0.73
Hypertension	1.00(1.00–1.00)	0.71
Educational level	1.02(0.98–1.07)	0.34
Income	1.00(1.00–1.00)	0.79
BMI	0.98(0.96–0.99)	0.01
Smoking	1.03(0.94–1.12)	0.53
Eating from common plate	1.00(0.95–1.06)	0.89
Use of hypoglycemic drugs	0.35(0.28–0.44)	<0.001
Insulin Use	0.12(0.09–0.15)	<0.001
Physical activity	1.00(1.00–1.00)	0.8
Constant	2.25	0.1

*The statistical significance was set as P–value ≤ 0.05*.

*The reference group in each variable was: Male (Gender); Primary school and below (Educational Level); No (Diet types); No(Dyslipidemia and Hypertension); Normal (BMI); Haven ever smoked (Smoking); No (Insulin and Hypoglycemic Drugs Use); Sitting most of the time (Physical Activity)*.

## Discussion

Our study aimed to identify a possible association between glycemic control and different diets followed by patients with type 2 diabetes. From the present study results, we found that a higher percentage of participants with poor glycemic control was in the age group between 51and 70 years old in all the followed diets. In one of their studies, Ma et al. (16) mentioned that aging changes the human body. This includes a decrease in the functions of some organs like the pancreas, a decrease in the response to insulin and decrease in glucose consumption with a decrease in muscle mass (16). Differences in the income levels of the two groups were also detected in our study participants. Our results showed that a higher proportion of university degree holders had HbA1c ≥ 7%. These findings are consistent with other studies in the literature (17–19). Several studies have shown that low levels of income may be related to an increased HbA1c since a higher income enables individuals to purchase different goods and services that will help them improve their health care. Patients with high-income high income also have lower chances of developing complications of diabetes, such as cardiovascular disease and retinopathy (18, 20, 21).

In this study, a low-salt diet was associated with poor glycemic control and increased waist circumference. These results, arguably, are the opposite of what is found in the literature and suggest that patients with these poor diabetic markers are motivated to follow a low-salt diet. To reach any conclusion, more research on medical advice and the desire to follow it is required. The following studies contradict our findings.

According to a survey that was done in Malaysia on Malaysian citizens aged 18 and above, the authors showed that sodium intake was higher among male participants with a higher waist circumference (22). Another study conducted to estimate the relationship between sodium intake and obesity revealed that sodium consumption was significantly associated with a larger waist circumference and greater BMI (23). Moreover, research done to investigate the relationship between urinary sodium and obesity indices found that greater urinary sodium excretion was associated with higher waist circumference besides other obesity markers (24). All the results confirmed the presence of a positive correlation between salt consumption and increasing waist circumference (22–24). Additionally, the present study showed an increase in the risk of poor glycemic control for patients, who were following a low-salt diet. Salt restriction increases insulin resistance (25, 26), which increases blood glucose levels and, thus, HbA1c. It has been shown that short-term sodium restriction stimulates the sympathetic nervous system to increase the production of blood catecholamine concentrations, thus, increasing insulin resistance (26). Urinary salt excretion is associated with glucosuria. As a result, when salt is restricted, the amount of glucose excreted in the urine decrease, resulting an increasing in blood glucose and HbA1c concentrations (27). Gress et al. (28) revealed that low sodium intake based on 24-h dietary recall was accompanied by a higher consultation of sugar (28). The negative impact of a low sodium diet on glycemic control may be explained at least partially by the associated high sugar intake (28). In agreement with our findings, two studies revealed that low fat intake reduces waist circumference and weight (29, 30). Hooper et al. (29) conducted a trial where participants were randomized to high-fat intake or low-fat intake with no intention of losing weight and found that participants with low-fat intake had a smaller waist circumference as compared to participants who were on a high-fat diet (27).

Many studies contradicted our results and showed that a low-fat diet is associated with better glycemic control. However, the results of the present study show that a low-fat diet increases the odds of poor glycemic control. A randomized control trial conducted by Kahleova et al. reported that a low-fat plant-based dietary intervention increased insulin sensitivity (31). Moreover, another study examined the effect of a low-fat vegan diet on insulin resistance and found a reduction in insulin resistance with a diet high in polyunsaturated fatty acids, particularly α-linolenic and linoleic acids (32). Our results may have a confounding effect where participants following the low-fat diet had high-sugar consumption as low-fat products are higher in sugar content than regular food products (33).

A study comparing the effect of a high-unrefined carbohydrate, low-fat diet (HC) on glycemic control with those of a very low-carbohydrate, and high-unsaturated/low-saturated fat diet (LC) found that both energy-reduced LC and HC diets with low-saturated fat content produce significant improvements in glycemic control in adults with T2DM. Glycemic control was greatest with the LC compared with HC (34). These results explain why patients who were following more than one diet had better glycemic control than those following one diet.

The main limitation is the determination of the diet type, which was based on the food choices and the participant's perception of his/her diet as compared to before diagnosis. Diet alone could not be considered the main reason for poor glycemic control. Other confounders can play a role in glycemic control, such as age, gender, and medication. Additionally, our study is a secondary analysis from a single time point survey and not a pre/post special diet. However, this study has several strengths. First, the sample size is a total of 2,448 participants, which represents a large number of the Qatari population. Additionally, the sample size is homogeneous since all patients are of Qatari nationality or have had a long-term residency in Qatar.

## Conclusion

The findings of this secondary analysis study suggested that patients with diabetes who stated that they regulated multiple factors of their diet (following a low-calorie, low-salt, and/or low-fat diet) may have better glycemic control. Longitudinal studies are needed to confirm our results.

## Data Availability Statement

The raw data supporting the conclusions of this article will be made available by the authors, without undue reservation.

## Ethics Statement

The studies involving human participants were reviewed and approved by Qatar BioBank. The patients/participants provided their written informed consent to participate in this study. The Institutional Review Board (IRB) of QBB approved under the IRB exempted category (Ex-2021-QF-QBB-RES-ACC-00058-180).

## Author Contributions

RT and HB were responsible for the study conception and design and responsible for the development of the methodology. RT and ZS were responsible for the analysis and interpretation of data. RT, ZS, AH, YE, MA-A, KA, and ZA-H were responsible for drafting the manuscript. RT, ZS, AH, and HB were responsible for reviewing and revising the manuscript. All authors contributed to the article and approved the submitted version.

## Funding

This article was supported by Qatar university Internal Grant No. QUEST-1-CHS-2022-280. The findings achieved herein are solely the responsibility of the author[s].

## Conflict of Interest

The authors declare that the research was conducted in the absence of any commercial or financial relationships that could be construed as a potential conflict of interest.

## Publisher's Note

All claims expressed in this article are solely those of the authors and do not necessarily represent those of their affiliated organizations, or those of the publisher, the editors and the reviewers. Any product that may be evaluated in this article, or claim that may be made by its manufacturer, is not guaranteed or endorsed by the publisher.
